# Post-weaning high-fat diet results in growth cartilage lesions in young male rats

**DOI:** 10.1371/journal.pone.0188411

**Published:** 2017-11-22

**Authors:** Samuel S. Haysom, Mark H. Vickers, Lennex H. Yu, Clare M. Reynolds, Elwyn C. Firth, Sue R. McGlashan

**Affiliations:** 1 Department of Anatomy and Medical Imaging, University of Auckland, Auckland, New Zealand; 2 Liggins Institute and Gravida: National Centre for Growth and Development, University of Auckland, Auckland, New Zealand; 3 Department of Exercise Sciences, University of Auckland, Auckland, New Zealand; Universidade do Estado do Rio de Janeiro, BRAZIL

## Abstract

To determine if a high-fat diet (HF) from weaning would result in a pro-inflammatory state and affect joint cartilage, we fed male rats either HF or Chow diet post-weaning, and voluntary wheel exercise (EX) or cage only activity (SED) after 9 weeks of age. At 17 weeks body composition, plasma biomarkers and histomorphology scores of femoro-tibial cartilages of HF-SED, HF-EX, Chow-SED and Chow-EX groups were compared. Food intake and activity were not significantly different between groups. HF diet resulted in significantly higher weight gain, %fat, fat:lean ratio, and plasma leptin, insulin and TNFα concentrations, with significant interactions between diet and exercise. No abnormal features were detected in the hyaline articular cartilage or in the metaphyseal growth plate in all four groups. However, collagen type X- positive regions of retained epiphyseal growth cartilage (EGC) was present in all HF-fed animals and significantly greater than that observed in Chow-fed sedentary rats. Most lesions were located in the lateral posterior aspect of the tibia and/or femur. The severity of lesions was greater in HF-fed animals. Although exercise had a significantly greater effect in reducing adiposity and associated systemic inflammation in HF-fed rats, it had no effect on lesion incidence or severity. Lesion incidence was also significantly associated with indices of obesity and plasma markers of chronic inflammation. Clinically, EGC lesions induced by HF feeding in rats from very early in life, and possibly by insufficient activity, is typical of osteochondrosis in animals. Such lesions may be the precursor of juvenile osteochondritis dissecans requiring surgery in children/adolescents, conservative management of which could benefit from improved understanding of early changes in cellular and gene expression.

## Introduction

In recent years, juvenile obesity has become alarmingly common, and several musculoskeletal diseases have been associated with higher prevalence in obese compared to non-obese children and adolescents [1–3]. The presence of some common musculoskeletal disorders in childhood is a significant risk factor for orthopaedic disease later in life. For instance, childhood fracture increases risk of fractures later in life [4, 5]. Obese children have a high risk of becoming obese adults [6, 7]. Obesity is a significant risk factor for osteoarthritis (OA) in adults, and is associated with complex interactions of chronic inflammation (both systemically and locally within the joint), the state of gut microbiota, and the immune dysfunction [8–10]. Adolescents who are overweight or obese complain of joint pain, and are less mobile [11, 12], and less prone to vigorous activity during childhood. Osteoarthritis may be a morbidity associated with obesity in the young, and is now included as a component of the Metabolic Syndrome [13], the prevalence of which is increasing [14].

Musculoskeletal diseases have been well documented descriptively at the time of end-stage disease, but are difficult to study at, or before onset of early-stage chronic disease. This is especially applicable in joint disease in the overweight or obese state which is typified by increased concentrations of plasma adipokines and inflammatory cytokines, some of which are known to affect cartilage [15–17]. The full consequences of the juvenile obese state on musculoskeletal function remain obscure at the mechanistic level, especially obesity during the period of growth and development. Although systemic and local joint adipokine and inflammatory cytokines are implicated in the pathogenesis of OA (reviewed in [8, 15]), studies have been confined to older animals [17, 18]. However, the effects of cytokines on the cartilages in the developing joint (hyaline articular cartilage and epiphyseal growth cartilage (EGC), sometimes referred to as the articular-epiphyseal-cartilage-complex [19] or the effects of physical activity at young ages have not been intensively studied.

In the present study we examined 4-month-old male rats derived from normal pregnancies to determine if a high-fat (HF) diet introduced at weaning would result in diet-induced obesity resulting in abnormal plasma inflammatory biomarkers and morphology of the femoral and tibial hyaline articular and growth cartilages. We also hypothesised that voluntary running wheel physical activity would have a significant effect on adiposity and commitment protective effects on cartilage health.

## Materials and methods

### Animals

All facilities, procedures and conduct of the study were approved by the Animal Ethics Committee of the University of Auckland. Male Sprague-Dawley weanling rats (n = 40, aged 21 days—d21) were housed in pairs, in standard 40.6 x 50.8 x 20.9 cm cages, maintained at 25°C and 12/12h light:dark cycle. Rats were sourced from the Vernon Janson Animal Unit, The University of Auckland. Bodyweight and food consumption were recorded twice weekly, and water was freely available. From d21 to d63, rats had *ad libitum* access to either a high fat (HF) diet (45% kcal from fat, D12451; Research Diets, Inc., New Brunswick, NJ), or a control Chow diet (18% kcal from fat, 2018 Harlan Teklad, United Kingdom). From d63 –d121, half of each diet group had only spontaneous cage activity exercise (SED), while the other could also voluntarily exercise on a running wheel. This yielded 4 groups in a balanced design (n = 10 per group) fed either chow or HF diet, with or without 8 weeks exercise from d63-65 to d121, referred to below as HF-SED, HF-Ex, Chow-SED, and Chow-Ex.

### Wheel exercise recording

Activity of rat pairs was recorded for 24 hours on a single day on or near d70, 80, 90, 100, and d110, and the running wheel exercise activity (distance run in metres) was recorded every 15 minutes (Lafayette Instrument Company, Lafayette, IN), except during brief procedures such as cage cleaning, and food/water provision. The data showed that 96.2% of the total exercise activity of both exercise groups was accrued between 6pm and 6am, and thus analysis was conducted on only the dark period data, after subtracting the short intervals of light period activity from the total 24 hour distance. Cumulative running distance was calculated from the 5 measurement periods per cage for each group. Spontaneous movement by pairs of rats within the cages was not measured.

### Body composition and body size measures

Dual energy X-ray absorptiometry (DXA) scanning (Lunar Prodigy, GE, Waltham, USA) was conducted on d117, under light isofluorane anaesthesia with the rat in sternal recumbency. Body composition (fat and lean mass) was calculated using dedicated animal software (Lunar Prodigy, Madison, USA). On d119-122 rats were fasted overnight and killed by decapitation following anaesthesia with sodium pentobarbitone (60 mg/kg, IP). The left femur, with all ligaments and tendons removed was measured using a digital caliper.

### Blood plasma biomarkers

Blood was immediately collected into EDTA vacutainers, kept on ice until centrifugation for 15 min at 3000*g* and plasma supernatant stored at -80°C for biomarker analysis. Leptin and insulin concentration were determined using commercially available rat-specific ELISA (CrystalChem, IL, USA, Cat#90040 and #90060, respectively), and interleukin (IL)1β and IL6 using a rat-specific ELISA (R&D Systems MN, USA; RLB00, R6000B), according to manufacturer’s instructions. IL1α, IL10, interferon (IFN)γ and tumour necrosis factor (TNF)α were measured using the BD^™^ Cytokine Bead Array (BD Bioscience, Auckland, New Zealand), a BD Accuri C6 Flow Cytometer and BD Accuri C6 Software. Cytokine concentrations were calculated using FCAP Array Software v3. All values obtained were above the minimal detectable limit.

### Femoro-tibial joint (FTJ) histology and grading

Following transection through right distal femur and proximal tibia, each whole femoro-tibial joint (FTJ) was collected within 10 minutes and placed in 4% paraformaldehyde for 48 hours at room temperature, and then washed 3 times for 10 minutes in 0.1M phosphate-buffered saline (PBS). Joints were decalcified in (35%) formic acid/ formate buffered solution for 13 days at room temperature prior to standard histological processing into wax. Sagittal sections (8μm) were collected every 200 μm from the entire joint and mounted onto poly-L-lysine coated slides. A reference section from both the medial and lateral condyles were determined using the following features: proximal extent of anterior and posterior femoral HAC at approximately the same level, no connection of anterior and posterior meniscal horns, no tibial or femoral cruciate ligament attachments visible in the section. The section 400μm lateral and 400μm medial to each of the two reference sections were selected for study, resulting in a total of 6 sections per joint. Sections were stained with toluidine blue, and imaged using a MetaSystems VSlide slide scanner (Version 2.1.124) running Metafer4 (version 3.9.2) with a 20X (NA 0.9) objective. Images were viewed using MetaSystems VS Viewer software (Version 2.0).

**Fig 1:**
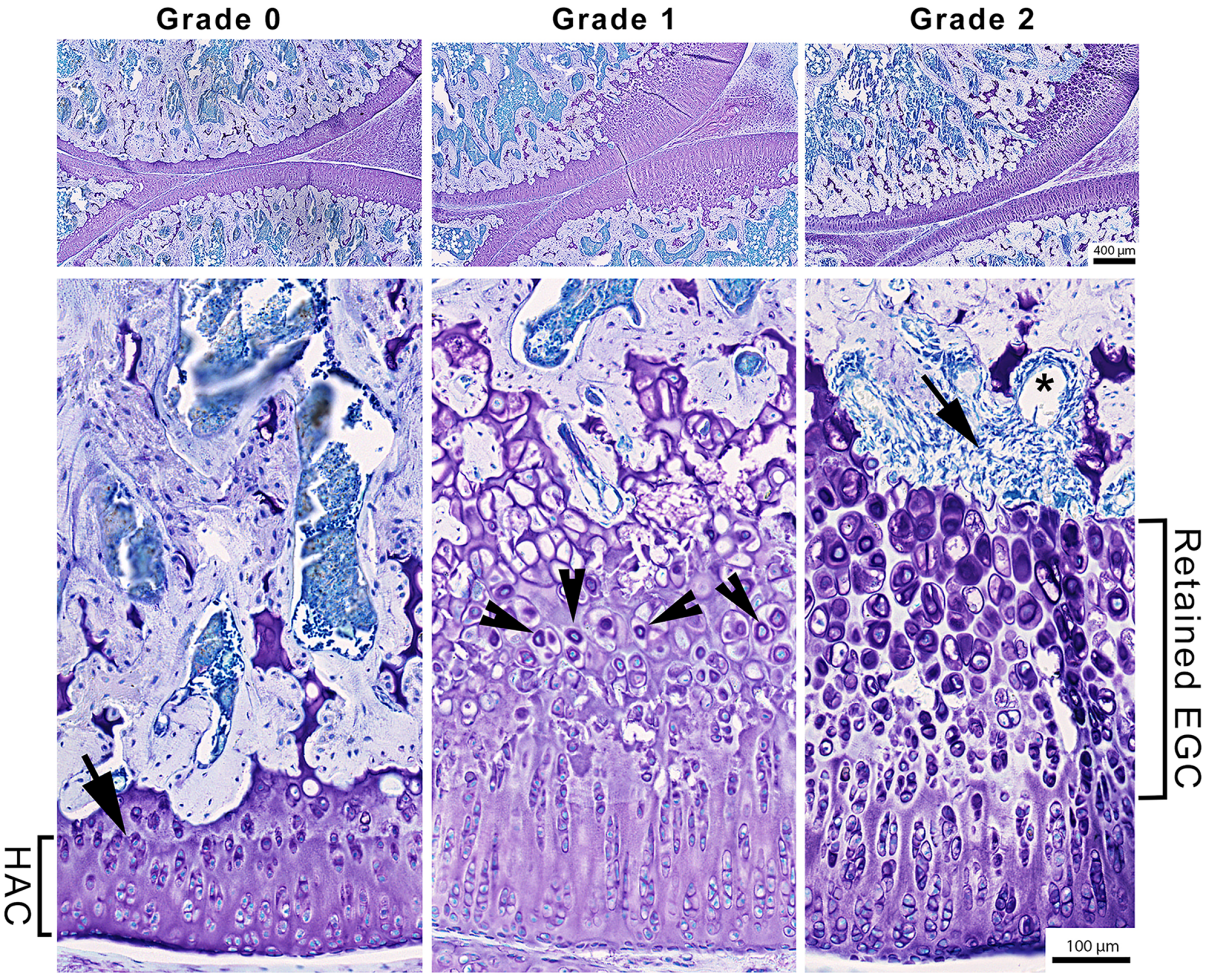
Representative toluidine-stained sagittal knee sections illustrating distal femoral epiphyseal cartilage graded 0, 1, 2 using a modified scoring detailed in Table 1. Grade 0 shows hyaline articular cartilage (HAC) and tidemark (black arrow). Grades 1and 2 show retained epiphyseal growth cartilage (EGC) containing hypertrophic cells with toluidine blue stained lacunae (arrowheads; Grade 1), and in grade 2 fibrosis, stained blue, is present between the retained EPG and the subchondral bone (black arrow) and a cavity/ cyst within the fibrotic tissue (*). Grade 3 lesions were not detected in this study.

A separate scoring system (Table 1), refined from a previous classification [20], was devised to score morphological changes in the EPG which are presented in Fig 1. A selection of 72 slides were scored on 2 separate occasions by two blinded investigators (SM and LY) and showed intra–rater agreement of 99.3% and inter-rater agreement of 95.1%.

**Table 1:** Epiphyseal growth cartilage grading system.

Grade	Essential Features	Additional Features
**0**	Consistent thickness of EGC Along the length of HAC: Consistent, roughly vertical zonal organisation of chondrocytes Tightly defined thin tidemark visible Consistent thickness of the ACC, which follows contours of underlying bone.	
**1**	EGC is focally thickened–extending into subjacent epiphysis Retained EGC characterised by: Chondrocyte hypertrophy Irregular chondrocyte organisation Reduced ECM volume Tidemark partially or completely indiscernible	Infiltration of toluidine blue stain into chondrocyte lacunae in retained EGC. Small voids or cavities formed between chondrocytes in the retained EGC.
**2**	Fissure(s) at chondro-osseous junction or within retained EGC. Fissures are either: Continuous along the entire length of the retained EGC **or**, Multiple fissures of shorter lengths **and/or**, Fibrous tissue in SCB zone, in regions subjacent to fissure(s).	
**3**	Fissure(s) at chondro-osseous junction or within retained EGC, are continuous through to the HAC surface (Kato et al., 1984), forming: A flap of HAC, ACC and EGC tissue, that projects into the joint cavity **or**, Complete separation of flap comprising HAC, ACC and EGC, forming a loose body.	Fibrous tissue or cyst-like lesion(s) in epiphysis, or subjacent to the flap or cleft.

HAC—Hyaline articular cartilage, ACC—Articular calcified cartilage, SCB—Subchondral bone, ECM = Extracellular matrix

The grades and associated morphological features of epiphyseal growth cartilage (EGC) from [20]. Additional features which are common to each grade are also listed. A particular grade required all the essential features listed.

Each of the six FTJ images from each animal was scored in the 4 joint regions: medial femoral condyle, lateral femoral condyle, medial tibial plateau, lateral tibial plateau used in the scoring system. The number of rats with one or more lesions was expressed as a percentage. The assigned grade from all 6 sections per joint was summed to determine the extent and severity of lesions within the entire joint. We also investigated if lesions were more commonly located on the medial or lateral aspects of both the tibia and femur.

### Collagen type X immunohistochemistry

Immunohistochemical analysis was conducted on grade 1 lesions from all groups (using the next serial section 200μm away). Grade 0 sections were used as controls. Paraffin-embedded sections were de-waxed, incubated in testicular hyaluronidase (2mg/ml) for 1 hour at 37°C, rinsed in distilled water prior to labelling with mouse monoclonal collagen type X antibody (1:200; clone X53, eBioscience Inc, USA) for 2 hours at room temperature, then detected using the Mouse-on-Rat HRP Polymer Bundle kit (Biocare Medical, USA), according to the manufacturer instructions. Sections were counterstained with hematoxylin, dehydrated and mounted in DPX.

### Statistical analysis

All data were tested for normality, and between-group differences were analysed by two-way factorial ANOVA, with diet and exercise as factors (SPSS, USA). To determine if the presence of a lesion was associated with a specific body parameter, % body fat, lean: mass ratio or plasma markers of adiposity and inflammation, the mean and SEM was calculated for rats with and without a lesion and compared using Mann-Whitney U test. All data is expressed as mean and standard error of the mean (SEM). P < 0.05 was considered statistically significant.

## Results

### Body weight, composition and exercise

**Fig 2:**
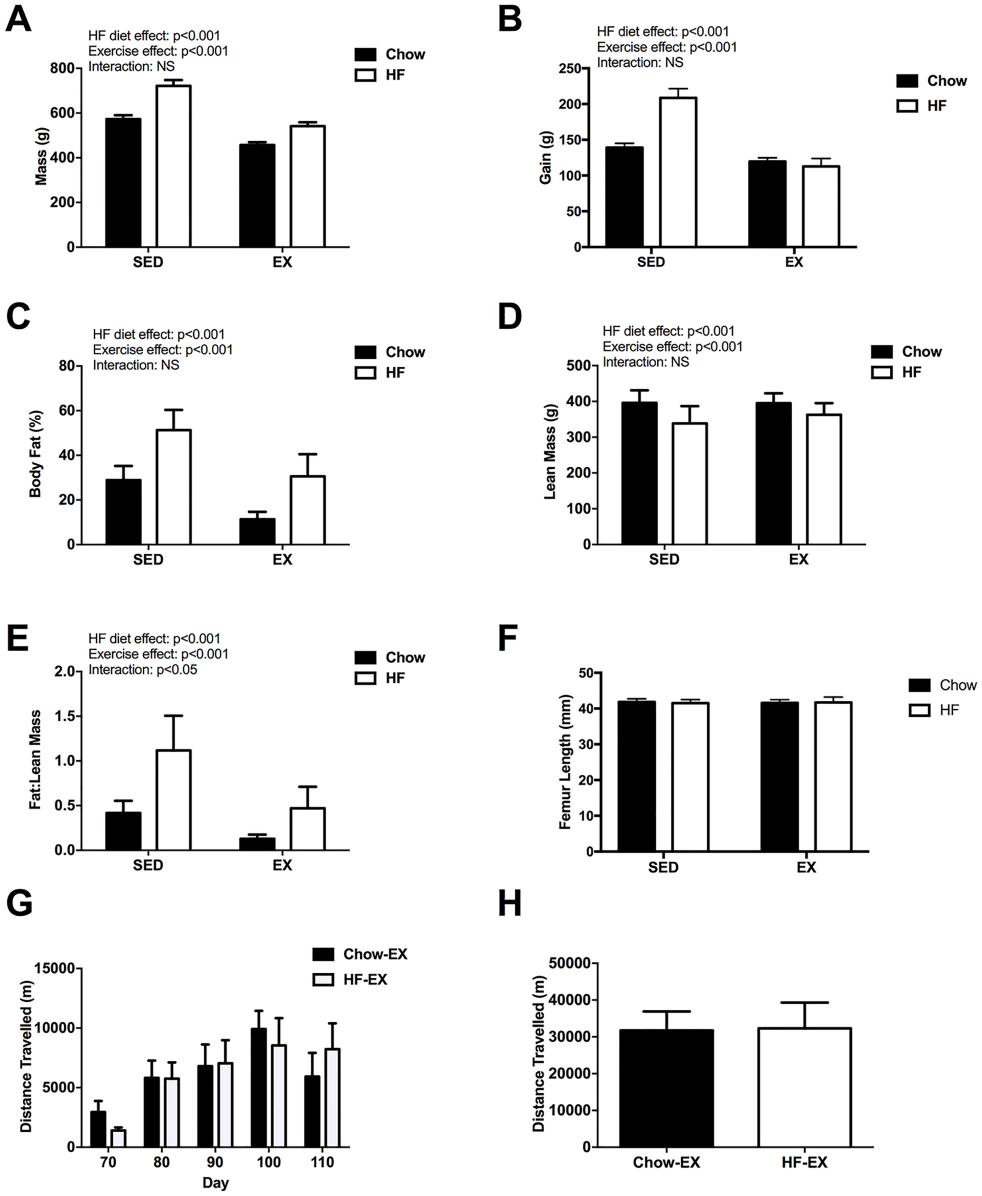
Diet and exercise changes in body parameters on d117 rats. (A) body mass (g), (B) gain (g) from d63-d121, (C) total body fat (%) (D) lean mass (g) (E) fat:lean ratio, and (F) femur length (mm). Data represents the mean (±SEM) from n = 10 rats/ group. Exercise data: (G) distance travelled (m) on d70, 80, 90, 100 and 110 in Chow and HF pairs and, (F) total distance travelled over the exercise intervention period. Exercise data (G, H) represents data obtained from running wheel per cage (2 animals/ per cage).

There was a significant direct effect (*P*<0.001) of HF diet on bodyweight at d117, being higher in the HF than the chow-fed groups; the effect of exercise on body weight was of similar significance and body weight was lower in wheel-exercised than control rats; the diet-exercise interaction on body weight was not significant. After introduction of wheel exercise, the increase in body weight by d117 was significantly greater in the HF-SED than in other groups (Fig 2A). For the exercise period, d63-d117, diet had a significant effect on weight gain (g) in Chow-SED rats (p < 0.001), and exercise mitigated the gain in the HF-EX rats compared to HF-SED group (Fig 2B). There was a significant interactive effect of diet and exercise (p < 0.05).

There were significant direct effects of HF diet and of wheel exercise on percentage body fat, lean mass and fat:lean mass ratio, which were higher in HF-fed than in Chow-fed rats (p <0.001), and lower in wheel-exercised than sedentary rats (p <0.001) (Fig 2C–2E). The diet-exercise interactive effect on body fat was not significant, but was significant (p <0.05) for fat: lean ratio, with exercise having a greater effect on fat:lean ratio in HF-fed than in chow-fed rats (Fig 2E). Diet and/ or exercise did not affect femur length (Fig 2F).

Mean running distance in both exercised groups gradually increased up to d100, with the lowest distance travelled on d70 as rats acclimatised to the wheel. There were no differences between Chow-EX and HF-EX on any of the days measured or in the overall total distance travelled (summed mean activity on the five measurement days) between exercised groups (Fig 2G and 2H).

### Cartilage morphology and grading

**Fig 3:**
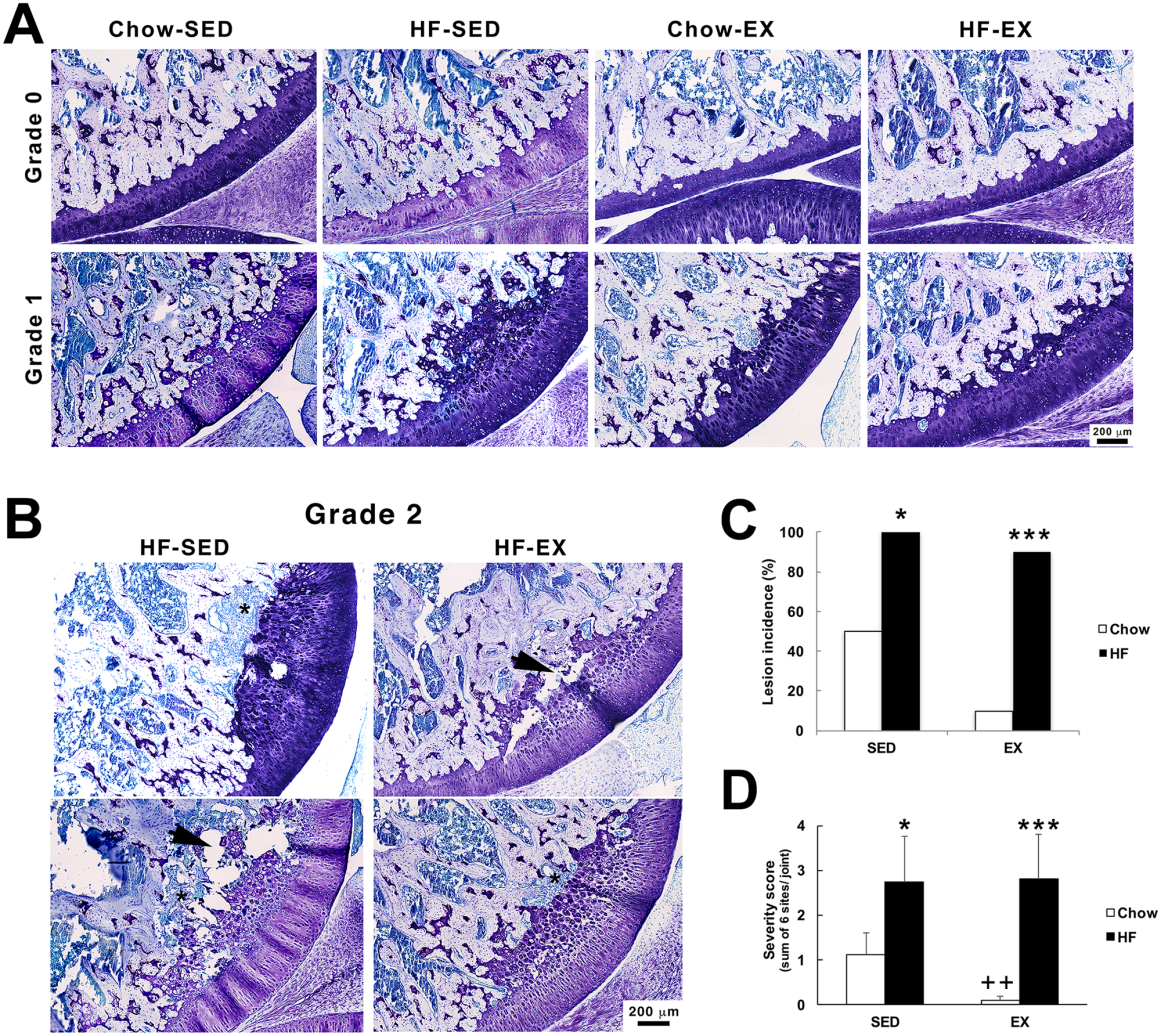
(A) Representative sections of Grade 0 and Grade 1 lesions from all 4 groups. (B) Grade 2 lesions were detected in high fat-fed rats only (total of 4 lesions detected) * indicates regions of fibrosis at the interface of the subchondral bone and EPG lesion; arrows indicate fissures within the lesion. (C) Incidence (%) of rats in which one or more lesions were detected. (D) Mean (±SEM) severity score (sum of grades from all 6 sites examined in each joint). n = 8 for SED groups, n = 10 for EX groups. * p < 0.05, **p < 0.01, ***p < 0.001. * indicates comparisons between chow and HF diets and + indicates comparisons between exercised groups.

Using the OARSI scoring guidelines for the rat FTJ [21], no abnormality was detected in the contour or surface integrity of the articular cartilage; there was no evidence of matrix depletion in the HAC. Grade 0 joint tissues displayed a consistent thickness across the joint margin and were thinner in anterior regions than posteriorly. The HAC exhibited a regular tightly defined tidemark, regular chondrocyte arrangement, and the calcified cartilage followed the contours of the subjacent bone (Fig 3A). Grade 1 cartilage contained thickened regions of toluidine blue-stained EGC directly subjacent to the HAC. The HAC showed uniform toluidine staining and regular zonal arrangement of chondrocytes, similar to Grade 0 cartilage. However, the tidemark was disrupted and present only intermittently over the width of the retained EGC region, where chondrocytes were hypertrophic within lacunae inflitrated with toluidine blue staining. The chondro-osseous junction followed the contours of the EGC which projected irregularly into the underlying subchondral bone (Fig 3A).

Grade 2 lesions were detected only in rats exposed to a HF diet (total of 4 animals). The lesions showed similar morphological characteristics to Grade 1 sections but also contained small and large fissures, fibrous tissue and occasional cavities or cysts (Fig 3B). No grade 3 lesions were detected in any of the sections analysed. All were in the femur, three in the postero-lateral aspect and one in the postero-medial apsect, of each joint. (Fig 3B). Twenty two of the 36 joints examined had one or more lesions present, and the incidence was significantly greater in HF-SED compared to Chow-SED (p < 0.05) and in HF-EX compared to Chow-EX rats (p < 0.001). Fifty percent of rats in the Chow-SED group had Grade 1 lesions present, although the extent to which they were found across the entire joint (severity score) was significantly less than in HF-SED rats (p < 0.05). Exercise significantly reduced the number and severity of lesions in the Chow-fed animals (p < 0.01) but had no effect in HF-fed rats where lesion incidence and severity were not significantly different between HF-SED and HF-EX groups (p >0.05).

A greater number of lesions were detected in the tibia (57%) compared to the femur (43%), the majority of which were located posteriorly, adjacent to the posterior horn of the lateral or medial meniscus. Lesions were most common in the lateral tibia in all groups except Chow-EX, in which only one Grade 1 lesion, in the lateral femoral condyle, was identified.

**Fig 4:**
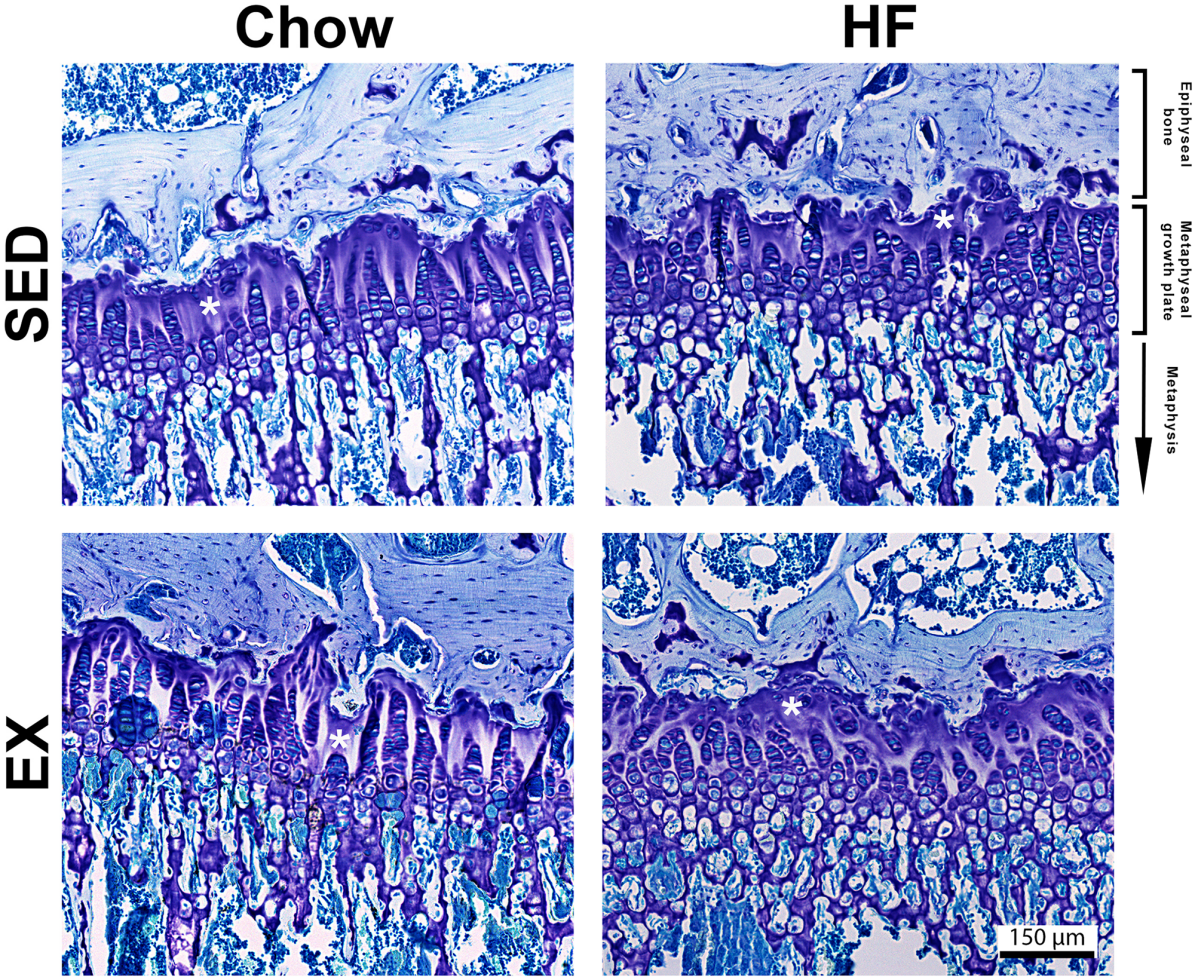
Representative toluidine stained sagittal sections of d117 metaphyseal growth plates from all four groups. No lesions or areas of retained growth cartilage were detected in any group. * indicates regions of acellularity, a common characteristic of physes of rats older than 16 weeks.

There was no evidence of retained growth cartilage in the metaphyseal growth plates (physes) (Fig 4). Tibial physes from each group showed consistent heights and displayed morphological characteristics of young adult rats. This included columns of proliferating cells (often narrowing at the proximal aspect towards the resting zone), with large areas of acellularity with ECM stained with toluidine blue, 4–5 rows of hypertrophic cells, and very few detectable resting cells.

### Collagen type X

**Fig 5:**
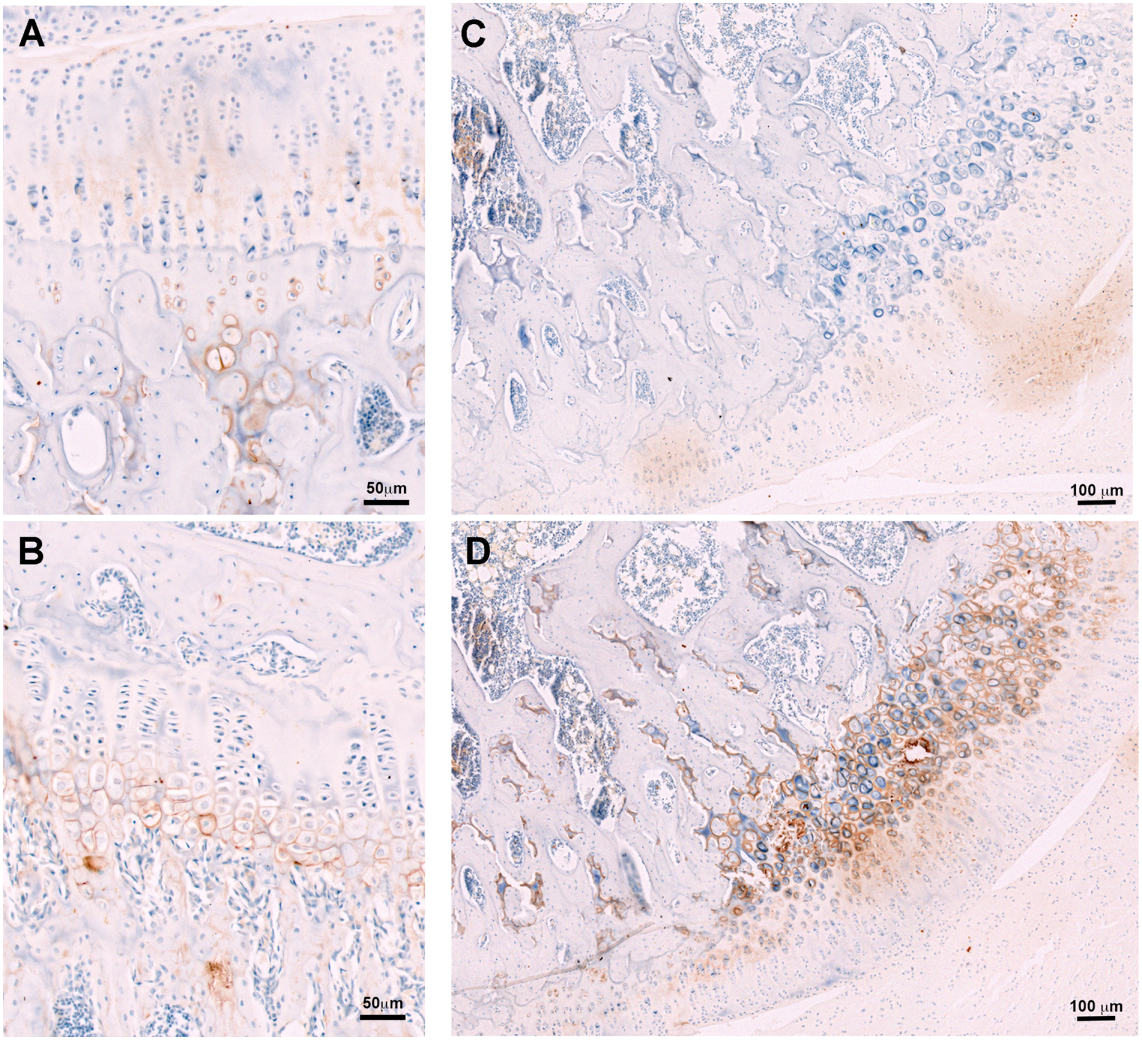
(A) Collagen type X labelling (brown) in the hyaline articular cartilage region in a section graded 0. Staining is specifically localised in hypertrophic cells below the tidemark. (B) Collagen type X distribution in the metaphyseal growth plate. (C) Negative control (no primary antibody) section of a Grade 1 lesion. (D) Collagen type X distribution within the retained epiphyseal growth cartilage.

To verify if the EGC lesions were due to retention of hypertrophic chondrocytes, grade 1 lesions were labelled with collagen type X antibody, and compared to Grade 0 sections. In healthy grade 0 sections, collagen X was present in only the pericellular zones surrounding the last 2–3 rows of hypertrophic chondrocytes, adjacent to the chondro-osseous junction (Fig 5A) and in the hypertrophic zone of the metaphyseal growth plate (Fig 5B). In contrast, in Grade 1 lesions, the retained EGC regions had intense collagen type X staining in pericellular regions of all cells within the lesion, which had up to 9 layers of hypertrophic chondrocytes (Fig 5D); Fig 5C shows a negative control image (no primary antibody) from a corresponding section.

### Plasma biomarkers

Mean fasting plasma leptin concentration was ~4.5 times higher in HF- than Chow-fed groups (p *<* 0.001), and about three times lower in EX than the SED groups (p < 0.001); the interaction between HF diet and exercise was significant (p *<* 0.024), where the effect of exercise on leptin concentrations was greater in the HF-fed than Chow-fed groups (Table 2). Similarly, mean fasting plasma insulin concentration was significantly altered (higher in HF-fed than Chow-fed groups, p *<* 0.009) and lower in wheel-exercised than SED groups (p <0.021), with no interaction effect. The effect of TNFα on diet and exercise was significant (p <0.003 and p <0.036, respectively), with higher concentrations in HF-fed than Chow-fed groups, and lower concentrations in EX than SED groups (Table 2). Mean IFNγ concentration was not influenced by diet, but was lower in EX than SED groups but only in Chow- and not HF-fed groups, with strong main and interaction effects (p *<* 0.006 and p *<* 0.007 respectively, Table 2).

**Table 2:** Plasma (mean ± SEM) cytokine (pg/ml) and adipokine concentrations (pg/mL) from each experimental group (n = 10/ group).

	Sedentary		Exercise		Diet	Exercise	Interaction
	Chow	High fat	Chow	High fat			
**Leptin**	5.91 (0.86)	27.26 (5.89)	1.86 (0.37)	8.61 (1.68)	p<0.001	p<0.001	p = 0.024
**Insulin**	1.83 (0.46)	4.12 (1.02)	0.91 (0.16)	2.04 (0.51)	p = 0.009	p = 0.021	NS
**IL-1α**	7.69 (0.17)	7.68 (0.23)	8.16 (0.56)	7.86 (0.30)	NS	NS	NS
**IL-1β**	9.89 (4.14)	14.34 (3.27)	8.56 (1.75)	15.98 (10.65)	NS	NS	NS
**IL-6**	51.92 (7.52)	43.96 (3.68)	48.79 (2.94)	73.70 (10.91)	NS	NS	p = 0.025
**IL-10**	22.82 (2.13)	53.10 (22.72)	27.02 (6.98)	26.61 (3.73)	NS	NS	NS
**TNF-α**	0.79 (0.16)	1.78 (0.36)	0.60 (0.10)	1.02 (0.16)	p = 0.003	p = 0.036	NS
**IFN-γ**	15.32 (1.78)	11.61 (1.42)	6.18 (1.15)	11.53 (1.91)	NS	p = 0.006	p = 0.007

### Associations between lesions and HFD

Body fat (%), plasma leptin and TNFα concentrations were significantly increased (p < 0.03, 0.04 and 0.02, respectively) in rats with a lesion present compared to rats where no lesion was detected (Table 3). There were no significant differences in body mass, lean mass, insulin and all other plasma cytokines between groups with and without lesions.

**Table 3:** Mean (± SEM) body weight (g), body fat (%), lean mass (g), leptin (ng/ ml), insulin (ng/ml) and plasma cytokines (pg/ ml) in groups of animals with or without a lesion. Statistical significance of the association was assessed by Mann-Whitney U test. No lesion (n = 18) and lesion (n = 22) groups and * p < 0.05 was considered statistically significant.

	No Lesion	Lesion	P-value
**Mass (g)**	558.1 (± 85.9)	619.7 (± 117.4)	0.09
**Body Fat (%)**	24.6 (± 15.8)	35.45 (± 15.0)	0.03*
**Lean Mass (g)**	379.9 (± 43.2)	367.6 (± 42.7)	0.40
**Leptin**	7.4 (± 9.9)	13.8 (± 15.8)	0.04*
**Insulin**	2.0 (± 1.9)	2.4 (± 2.5)	0.30
**TNF-α**	0.8 (± 0.4)	1.3 (± 0.9)	0.02*
**IFN-γ**	9.9 (± 6.2)	12.2 (± 5.5)	0.25
**IL1-α**	7.9 (± 1.3)	7.8 (± 0.9)	0.70
**IL1-β**	12.7 (± 11.4)	11.8 (± 23.0)	0.25
**IL6**	49.6 (± 18.6)	58.9 (± 27.9)	0.19
**IL10**	25.5 (± 16.8)	38.0 (± 49.8)	0.18

## Discussion

HF diets have been associated with higher body weight and percentage body fat in rodent studies [17, 22–26]. In the present study a HF diet resulted in marked increases in total body fat and fat:lean ratio. Food intake was similar in all groups, indicating that diet composition and not simply food intake was the most likely influence affecting body weight. Body weights increased more in HF-fed than chow-fed groups, presumably because of dietary energy density; the higher bodyweight was largely due to the increased percentage of body fat compared to lean mass fraction, as evidenced by the higher fat:lean mass ratio. This overweight/obesity phenotype was associated with significant effects of diet on leptin (hyperleptinemia), insulin (hyperinsulinemia) and TNFα, one of the major pro-inflammatory mediators implicated in the chronic inflammatory state associated with the onset, maintenance and progression of the obesity phenotype.

Voluntary wheel exercise was associated with lower body weight and percentage body fat in both Chow and HF groups, and the effects were similar to those in previous rodent studies, independent of type and intensity of exercise regime [17, 24, 26–28]. The present study is the first to examine the effects of HF diet and exercise in young rats during the rapid growth phase, and we show that the exercise-related differences in body composition are very similar to those shown in older adult rats. Despite the mild level of the exercise, and the young age of the rats, the voluntary exercise also had significant positive effects on metabolic homeostasis, whereby biomarkers related to the obese state (leptin and insulin) and inflammatory cytokines showed a marked reduction compared to groups not exposed to wheel exercise.

In contrast to previous studies which employed HF diets with fat content ranging from 30–62% to induce metabolic osteoarthritis, we detected no morphological evidence of changes consistent with early osteoarthritis on careful examination of large numbers of sections in any of the four groups [16, 17, 29–34]. Previous studies have introduced a HF diet at 8–12 weeks of age compared to 3 weeks of age in this study, and maintained animals on the HF diet for longer periods (commonly for at least 12 weeks), and may be why we did not observe any changes consistent with OA [29–31, 35]. Further, the absence of OA lesions may also be because the rats were male (less active than females), and the obese and chronic inflammatory state induced was mild. A voluntary activity regimen was chosen to reduce the likelihood of very high numbers of cycles, high forces, or exercise-related injury, because some imposed regimens elevate the likelihood of stress responses [36].

The study shows that the HF diet fed to rats for 3 months from weaning resulted in a morphological abnormality in the EPG of the distal femur and proximal tibia. The changes in the EPG morphology were characteristic of osteochondrosis, previously reported by Kato [20, 37, 38]. The severity of disturbances in cartilage morphology were reasonably uniform, and consisted of a combination of a loss of the tidemark in the area of the disturbance, localised thickening of the EGC hypertrophic cell layer, variability in chondrocyte hypertrophy and columnar organisation, reduction in chondrocyte extracellular matrix (ECM) volume, and discontinuity at the chondro-osseous junction. This was supported by the presence of a marker of chondrocyte hypertrophy, type X collagen, which showed positive labelling within the ECG lesions [39, 40].

Almost immediately adjacent to the focal EGC morphological disturbance, and further along the joint contour to the periphery of the joint, the morphology and the micro-anatomical relationship of the EGC and HAC appeared normal. The EGC abnormality had not resulted in collapse, fissuring or flap formation at and adjacent to the focal site of epiphyseal cartilage retention. These are typically classified as “stable” lesions [41]. The lesion was at a consistent site at the junction of the posterior and middle third of the lateral and medial condyle in both the femur and tibia, which is the most common lesion site in juvenile osteochondrosis in humans [42].

Lesion incidence was associated with factors related to obesity, namely percentage body fat, plasma leptin concentration and markers associated with chronic inflammation (TNFα). The inference is that the EGC morphological disturbance was most strongly associated with the HF diet, which induced obvious but not morbid obesity, and a mild pro-inflammatory state. The EGC morphological disturbances in this study are typical of those found in very early disturbances of endochondral ossification in domestic animals, mainly dogs [43], pigs [44, 45], horses [19, 46, 47] and intensively raised birds (chickens and turkeys). Osteochondrosis in animals has been associated with high growth rate in particular stages of early life [48]. The morphological hallmark of the disease is retention of growth cartilage due to focal delay in endochondral ossification of two kinds, articular osteochondrosis when the EGC subjacent to the HAC is affected, and the so-called physeal form of osteochondrosis, in which retention of physeal cartilage occurs, most commonly on the metaphyseal aspect of the physis, although retention of EGC where it is apposed to the epiphyseal aspect of the physis can also occur. The disease can cause swelling of the joint or of metaphyseal bone, pain, and functional disturbance, but not in the early stages. Compromise or failure of vascular structures within the growth cartilage is thought to be the most likely initiating factor of the EPG morphological abnormality [49–51].

Exercise had little causative effect on osteochondrosis lesion frequency in a major longitudinal study of young foals, but influenced lesion appearance and distribution; by contrast lack of exercise in early life was associated with more severe lesions [52]. This concurs with our current study, which shows that spontaneous wheel exercise had a significant direct effect on rat body composition, but almost no effect on the number of lesions in the HF-EX group compared to the HF-SED group, despite the exercise being associated with fewer lesions in the Chow-fed group. The lesions we describe appear to be similar to those seen in production animals, although we did not observe severe lesions seen in older rats [38], and the prevalence of mild lesions in our study was far higher due most likely to *ad lib* feeding of high-energy diet.

The appearance of lesions within the epiphyseal growth cartilage and not in the metaphyseal growth plate also supports that the lesions are typical of osteochondrosis. Neither HF diet nor exercise regimes had any effect on the overall growth of the rats, with no differences in femur length. Furthermore, the metaphyseal growth plate at d117 showed no major histopathology or difference in height or cellular organisation [53].

Osteochondrosis is not uncommon in some groups of young people, is clinically silent in the early stage of the disease (as it is in animals) [54], but robust characterisation of the early cartilage abnormality in humans is not accurately described [55]. The naturally-occurring osteochondrosis models in animals may be highly useful in assessing factors important in pathogenesis, attenuation or prevention of the human disease, and is surprising that the disease, previously described in rats [20] has not been followed up in more recent studies. One of the limitations of this study was the time course. Further study is required to determine if the cartilage lesions are self-resolving in rats. We had suspected that evidence of early OA would be present in rats in which a chronic inflammatory state was reached, but no changes in the hyaline cartilage surface, cells or matrix were observed. This is consistent with findings in human juvenile osteochondrosis [41], which is not usually associated with overweight or obese body type, although data on history of overweight phenotype at earlier life stages and chronic inflammatory status is lacking.

We presume that the amount and nature of some component of the HF diet was associated indirectly with the presence of the endochondral ossification disturbance. Because the rats were not subject to imposed exercise it seems unlikely that physical activity played a role. Further investigation of individual rats’ propensity to develop and maintain (or resolve) such lesions are required, concentrating on determining if EGC lesions can be reliably induced, if they persist throughout adulthood, if they result in premature OA through effects on the adjacent HAC (which is possible in children/adolescents [42]), and what particular features of lipid diets might lead to the presence of lesions in epiphyseal cartilage.

## Conclusions

In conclusion, we have demonstrated, for the first time, that exposure to a HF diet from weaning results in significant lesions in the EGC, typical of articular osteochondrosis. Our results demonstrate that the disturbance in endochondral ossification at the osteochondral junction is strongly correlated with body fat, plasma leptin and plasma TNFα concentrations, and not with exercise. A background of mild lesion incidence in chow-fed animals suggests that lack of exercise is a risk factor. The cellular and molecular mechanism of osteochondrosis is not fully understood in any species, and the relative contribution of body weight, growth rate, physical activity, nutrition and chronic inflammatory state are disputed. Further, molecular or cellular studies in juvenile human tissue are limited. Our findings highlight the sensitivity of the developing growth cartilage to high-lipid feeding, and contribute an additional potential mechanism involved in the pathogenesis of osteochondrosis. The model offers a unique opportunity to determine possible strategies for the prevention or progression of early stable juvenile osteochondrosis in children and adolescents.

## Supporting information

S1 Table Raw data values. All data values obtained from plasma, DXA and osteochondrosis measurements from each experimental group (n = 40 animals, N = 4 groups).
(XLSX)
